# Phylogenetic Study of the Evolution of PEP-Carboxykinase

**Published:** 2007-12-11

**Authors:** Sanjukta Aich, Louis T.J. Delbaere

**Affiliations:** Department of Biochemistry, University of Saskatchewan, Saskatoon, Saskatchewan, S7N 5E5, Canada

**Keywords:** PEP-carboxykinase, evolution, phylogenetics

## Abstract

Phosphoenolpyruvate carboxykinase (PCK) is the key enzyme to initiate the gluconeogenic pathway in vertebrates, yeast, plants and most bacteria. Nucleotide specificity divided all PCKs into two groups. All the eukaryotic mammalian and most archaeal PCKs are GTP-specific. Bacterial and fungal PCKs can be ATP-or GTP-specific but all plant PCKs are ATP-specific. Amino acid sequence alignment of PCK enzymes shows that the nucleotide binding sites are somewhat conserved within each class with few exceptions that do not have any clear ATP- or GTP-specific binding motif. Although the active site residues are mostly conserved in all PCKs, not much significant sequence homology persists between ATP- and GTP-dependent PCK enzymes. There is only one planctomycetes PCK enzyme (from *Cadidatus Kuenenia stuttgartiensis*) that shows sequence homology with both ATP-and GTP-dependent PCKs. Phylogenetic studies have been performed to understand the evolutionary relationship of various PCKs from different sources. Based on this study a flowchart of the evolution of PCK has been proposed.

## Introduction

Phosphoenolpyruvate carboxykinase (PCK), a carboxylase enzyme in nature, (EC 4.1.1.32 (GTP-dependent) or EC 1.1.49 (ATP-dependent)), is present in all known groups of living organisms. It catalyzes metal-nucleotide coupled reversible decarboxylation and phosphorylation between phosphoenolpyruvate (PEP) and oxaloacetate (OAA) depending on the system and the availability of the intermediates. In vertebrates, fungi, plants and in most bacteria, production of PEP from OAA by PCK is the key step for gluconeogenesis to produce glucose during fasting. In humans, increased gluoconeogenesis is responsible for the high blood glucose level in non-insulin-dependent diabetes mellitus (NIDDM) patients. Otherwise, in healthy people cytosolic PCK enzyme is only present during glucose starvation; cytosolic PCK rapidly disappears on the resupplying of glucose due to hormonal control of the transcription of the cytosolic PCK-gene. In some bacteria such as *Anaerobiospirillum succiniciproducens* (Cotelesage et al. 2005), parasitic helminthes like *Ascaris suum* (Rohrer et al. 1986), nematodes such as *Haemonchus contortus* (Klein et al. 1992), PCK carry out the reverse reaction to produce OAA from PEP. In kinetoplastid parasites, such as *Trypanosoma cruzi* (Trapani et al. 2001) and all species of the genus *Leishamania*, this enzyme is very active even in the presence of high levels of carbohydrate, producing a mixture of CO_2_, succinate and alanine as end products.

PCKs can be divided into two groups, based on its specificity towards the nucleotide substrate: ATP-dependent PCKs are mainly present in bacteria, yeast and plants, GTP-specific PCKs are mostly present in higher eukaryotes, most archaeons as well as in some bacteria (Fukuda et al. 2004). Fungal PCKs can be either ATP- or GTP-dependent. While there is significant sequence homology among each class, no statistically significance homology is found between the PCKs of the two classes. The structural studies from six different structures of PCKs, solved so far, demonstrated that the metal-binding and oxaloacetate binding active site residues are conserved in both ATP- and GTP-dependent PCKs (Holyoak and Nowak, 2006; Cotelesage et al. 2005; Dunten et al. 2002; Trapani et al. 2001; Matte et al. 1996; Sudom et al. 2001; Leduc et al. 2005). The nucleotide binding motifs are also almost conserved within each category but are unique for each class with few exceptions. The most archeon PCKs and *Giardia intestinalis* PCK have a unique GTP-binding region, compared to the GTP-binding motif present in other GTP-specific PCKs (Fukuda et al. 2004). It is interesting to know how and why two types of nucleotide specificities evolved.

The universal tree of evolution based on ribosomal RNA separates the three domains of archaea, bacteria and eukarya and places extreme thermophiles at the base of the bacteria (Brown et al. 2001). Another approach to make the universal tree based on protein sequence produces lots of mixing between domains. In spite of intermixing of the domains based on protein sequence homology, this method might be useful to understand the branching more accurately, as proteins control the cellular processes to maintain life. The three dimensional structure of a protein in its active form can provide important clues about how the protein performs its function. However structure determination of the protein is not always easy. Sequence alignment of similar proteins in distantly related organisms can also provide us with evolutionary relationships. Recent progress by genomic sequencing projects from a wide variety of species allows us to resolve more robust phylogenetic relationships among species.

Alignment of all PCK enzymes from the NCBI data bank shows some interesting results. In this paper we made the phylogenetic trees based on number of PCK proteins and genes sequences available in NCBI until 2007. To simplify the figure we only present a few selective species from all three domains of life. Extensive sequence alignment shows that the active site amino acid residues and metal binding sites (kinase 1a and kinase 2 regions) are almost but not completely conserved in PCKs of any origin and some have different nucleotide-binding sequences, which do not have any obvious specific nucleotide-binding motif for ATP or GTP. Based on protein sequence similarity and nucleotide specificities we propose a very simple evolutionary flowchart for PCK.

## Phylogenetic Analysis

**Figure 1.**
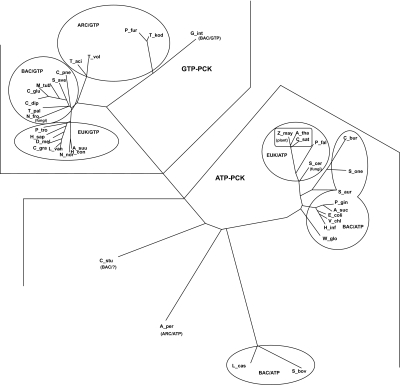
Phylogenetic tree based of amino acid sequences of few selected PCKs originated by the program PHYLIP, initially aligned with ClustalW.

Initial sequence alignment of all the species containing PCK proteins and genes were performed using ClustalW (Higgins et al. 1994). Prodist and Dnadist software programs under PHYLIP (Felsenstein, 1989) were used on the aligned sequences which produce a distance matrix file. Performing Neighbor-Joing and UGMA (unweighted mean) on the distance matrix file and lastly by employing Drawtree we produced an unrooted tree diagram for all PCKs to give the evolutionary relationship between the species (Felsenstein, 1989). The phylogenetic tree (Fig. 1) based on the enzyme sequences of PCKs, nicely divided ATP- and GTP-dependent PCKs into two regions. In each region of the tree, the PHYLIP program also grouped different species in the same branch or in proximity with very few exceptions, which are labeled. The archaeal *Aeropyrum pernix* (A-per) is placed close to other ATP-dependent bacterial PCKs, although it does not contain the conserved ATP-binding pockets (RX_5_TR) of bacterial or eukaryotic PCKs. At the NCBI data bank *Aeropyrum pernix* (A-per) (NCBI accession no: **Q9YG68**) is the only ATP-dependent PCK from archaea. A-per PCK shows less than 10% sequence homology with all other archaeal PCKs, which are GTP-specific but it shares more than 25% sequence homology (by bl2seq) with other ATP-specific PCKs from bacterial or eukaryotic origin (Table 1) (Tatusova and Madden, 1999). Another bacterial PCK from the group planctomycetes *Candidatus Kuenenia stuttgartiensis* (C-stu) (NCBI accession no: **CAJ75104**) is placed close to A-per PCK. C-stu PCK also does not have a conserved ATP-binding pocket. These two PCKs might have a conserved arginine in the ATP-binding pocket as shown in the active site alignment table (Table 2). Both of these PCKs from A-per and C-stu have 51% similar (positive) and 31% identical aminoacid sequences. C-stu PCK has 23%–36% amino acid sequence identity (by bl2seq) with various ATP- and GTP-specific PCKs from bacterial and eukaryotic species (Table 1). C-stu PCK does not have sequence similarity with other archaeal PCKs (except A-per) and with a few eukaryotic PCKs. A PCK from the archaon *Thermoplasma volcanium* (T-vol) (NCBI accession no: **P58306**), which contains a well conserved PCK-binding pocket like other GTP-dependent bacterial and eukaryotic species, also shows sequence similarity (23% identity, and 40% similarity) with C-stu PCK. The PCK from T-vol shows sequence similarity and identity with all other archaeal GTP-PCKs as well as all other GTP-dependent PCKs (Table 1). Another two GTP-dependent PCKs such as *Drosophila melanogaster* (NCBI accession no: **P20007**) and *Chasmagnathus granulate* (NCBI accession no: **AAL78163**) also have enzyme sequence homology with few other GTP-specific PCK enzymes similar to that of T-vol (Table 1).

In archea there are two distinct types of GTP-binding pocket; in one group they have a conserved GTP-binding pocket like other bacterial and eukaryotic PCKs (F/YXXXF/Y), while in the other few archea and in *Giardia intestinalis* (NCBI accession no: **AAG47713**), PCKs have distinct GTP-binding residues (Table 2). These archaea and *Giardia intestinalis*, PCKs also show more than 23% sequence identity with T-vol PCK.

In other two PCKs from *Lactobacillus casei* (NCBI accession no: **ZP_00386357**) and *Streptococcus bovis* ((NCBI accession no: **BAE46992**) the amino acid sequence of the ATP-binding pocket is not totally conserved (Table 2).

PCKs from *Nephrops norvegicus* (NCBI accession no: **CAB65311**) and *Litopenaeus vanamei* (NCBI accession no: **CAB85964**) do not have the lysine for the kinase 1a/P-loop (XKT) which is conserved in all other PCKs, but the threonine is conserved (Table 2). Alignment (Table 2) shows that in *Nephrops norvegicus,* the conserved glycine is also absent. In *Lactobacillus casei* and *Streptococcus bovis,* between lysine and threonine there is one serine residue. The conserved asparagine residue in this P-loop for GTP-specific PCKs is replaced by threonine in ATP-dependent PCKs. In the GTP-dependent PCK with a unique GTP-specific pocket, which is not conserved in other bacterial and eukaryotic PCKs, this asparagine is replaced by a serine residue. Similarly alignment studies also show that A-per PCK does not have the second lysine in its PCK-specific domain (XKK). The kinase 2 (XDD) domain is conserved in all PCKs where X in XDD is also glycine in most cases, with few exceptions where it is replaced by serine/histidine/glutamine. Initially we aligned all the PCK sequences available at NCBI and made the phylogenetic tree; later we chose a few from each domain group and the PCKs which have some importance (Table 2) to avoid overlap in the figure. The reactive cysteine residue (Cys 288 for human) which is conserved in most of the GTP-specific PCKs is replaced by serine in T-vol and *Thermoplasma acidophilum* (T-aci) PCKs (NCBI accession no: **Q9HLV2**) PCK.

**Figure 2.**
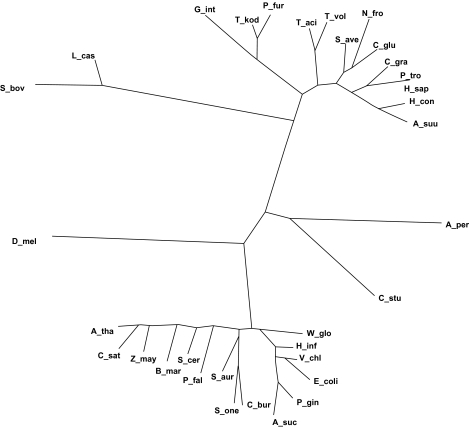
Phylogenetic tree based of DNA sequences of few selected PCKs originated by the program PHYLIP, initially aligned with ClustalW.

The phylogenetic tree based on PCK genes (Fig. 2) is partly similar to the tree obtained from PCK enzymes with few exceptions. A-per and C-stu PCKs are in the same branch. But D-mel, a GTP-dependent PCK is placed more closely to ATP-dependent PCKs. Also L-cas and S-bov, two ATP-dependent PCKs, were nicely placed in the same branch but more closely to the GTP-specific PCKs. Otherwise the program grouped all other similar PCKs closer just like other phylogenetic trees based on PCK enzymes.

The score resulted from ClustalW (Table 3) for PCK genes, representing the identity between two, is very inconclusive. The value is 10 between A-per and C-stu otherwise all values are less than 10 for these two PCK genes compared to other PCK genes. For D-mel PCK, the gene score value is also very low for all other PCKs. Other two GTP-dependent PCKs C-gra and T-vol show some high scores only with other GTP-dependent PCKs.

## Discussion Based on Phylogenetic Results and Sequence Alignments

The sequence alignment of all the PCKs demonstrate the presence of a PCK-specific domain (XKK), kinase 1a/P-loop (XKT), kinase 2 region (GDD) and nucleotide-specific pockets (Table 2). The catalytic site consists of the PCK-specific domain, the kinase 1a and kinase 2 regions which are almost conserved in ATP- and GTP-dependent PCKs from all organisms with very few exceptions as shown in the sequence alignment (Table 2). Sequence alignment of all PCKs available at NCBI shows us a few interesting features of some PCKs. The structural analysis from X-ray diffraction studies shows that the second lysine in the PCK-specific domain binds with divalent metal ion in the second metal binding site. The absence of the conserved second lysine residue in A-per PCK might eliminate the second metal ion binding site.

The lysine residue in GKT/P-loop (Lys 254 in *E. coli*) is interacting with both the β- and γ-phosphoryl groups of ATP. The positive charge of lysine helps in stabilizing the phosphoryl group during transfer (Matte et al. 2001). The absence of this lysine in N-nor and L-van might affect the reactivity of these enzymes. In this P-loop there is a reactive cysteine which is mostly conserved in all GTP-dependent PCKs. Reactive cystine, however, is replaced by serine in T-vol and T-aci which are two archeal PCKs with well defined GTP binding motif. It might be interesting to know how the absence of this cysteine can affect the stability of the P-loop in the GTP-dependent PCKs in T-vol and T-aci.

Although the phylogenetic tree (Fig. 1) constructed from selected important PCK enzymes is very similar to the tree made by Fukuda et al. where the emphasis was given on the position of archeal PCKs (Fukuda et al. 2004), the current phylogenetic tree is more extensive and elaborate. We have included few more PCKs from other species to explain the evolutionary origin of PCK. The tree reported here also shows that ATP- and GTP-dependent PCKs are present in all three major domains of life. All mammalian PCKs are GTP dependent, plant PCKs are ATP dependent and bacterial and fungal PCKs can be ATP- or GTP-dependent. It is interesting to note that parasitic nematodes, like *Ascaris suum* and *Haemonchus contortus* where PCKs preferentially carboxylate PEP to OAA, have been placed in a separate branch in the tree.

Many scientists hypothesize that the Archaea are the closest modern relatives of earth’s first living cells. They are called universal ancestors from which all other life is believed to have evolved (Woese, 2000). All archaeal PCKs are GTP-dependent except for *Aeropyrum pernix* (A-per). From amino acid sequence similarity, A-per PCK is closer to the ATP-binding PCKs (Table 1). It does not have any sequence similarity with other archaeal PCKs, which justifies its position in the phylogenetic tree (Fig. 1).

The most interesting PCK is bacterial planctomycetes *Candidatus Kuenenia stuttgartiensis* (C-stu) which placed close to A-per PCK. ATP-and GTP-specific PCKs have less than 10% sequence similarities, although C-stu PCK shows a very unique feature (Table 1). C-stu PCK has 23% – 36% amino acid sequence identity (by blast2) with various ATP- and GTP-specific PCKs from bacterial and eukaryotic origin (Table 1). This is the only PCK, so far, which has sequence similarity with both ATP- or GTP-binding PCKs but does not have any well defined nucleotide-binding motif. But still there is another valid point. C-stu PCK does not have sequence similarity with most other archaeal and few other GTP-dependent PCKs with only one exception (T-vol). The sequence homology between the archaeal PCK from T-vol and C-stu PCK might gave us the missing link. Sequence homology analysis also shows that other two GTP-specific PCKs, from D-mel and C-gra, have similar sequence homology with other GTP-specific PCKs and also with C-stu PCK but with any ATP-dependent PCKs. We can place C-stu PCK, which has sequence similarity with both ATP- and GTP-dependent PCKs) and does not have a very well recognized nucleotide-binding pocket, at the root (Table 2). Does it mean initially that PCKs did not have any ATP- or GTP-specificities, which have been acquired gradually with evolution? The position of plantomycetes is also very controversial in the phylogenetic tree. One recent analysis placed planctomycetes at the deep branching position near the root (Fuerst, 1995). Based on this concept and our homology studies we propose a very simplified evolution flowchart for PCK (Table 4). It would be interesting to know the active site residues and the tertiary structures of C-stu, T-vol and A-per PCKs to complement this evolutionary perspective.

## Figures and Tables

**Table 1.** Bl2seq shows sequence Identity (I) and homology (positive, P) between few selected PCK proteins from all domains of life.

**No**	**Organism**	***D_mel***		***C_gra***		***T_vol***		***A_per***		***C_stu***	

		**I**	**P**	**I**	**P**	**I**	**P**	**I**	**P**	**I**	**P**
1	*C_stu*	30	43	29	42	23	40	31	51	100	100
2	*D_mel (GTP)*	100	100	64	77	42	60	–	–	30	43
3	*C_gra (GTP)*	64	77	100	100	39	56	–	–	29	42
4	*T_vol (GTP)*	42	60	39	56	100	100	–	–	23	40
5	*A_per*	–	–	–	–	–	–	100	100	31	51
6	*A_suu (GTP)*	56	73	53	70	43	61	–	–	–	–
7	*H_con (GTP)*	55	71	53	71	41	61	–	–	–	–
8	*N_fro (GTP)*	50	68	45	64	40	58	–	–	–	–
9	*G_inf*	29	47	27	44	23	45	–	–	–	–
10	*P_fur*	29	48	30	48	31	49	–	–	–	–
11	*T_kod*	31	47	30	46	32	49	–	–	–	–
12	*S_ave (GTP)*	51	65	48	64	46	64	–	–	–	–
13	*T_aci (GTP)*	43	60	39	56	72	86	–	–	–	–
14	*P_tro (GTP)*	63	78	67	86	36	53	–	–	–	–
15	*H_sap (GTP)*	64	78	65	78	41	59	–	–	28	41
16	*L_van (GTP)*	67	78	75	84	40	57	–	–	28	41
17	*C_glu (GTP)*	48	65	44	62	44	61	–	–	28	43
18	*C_pne (GTP)*	51	66	47	65	46	62	–	–	28	45
19	*T_pal (GTP)*	52	67	47	62	45	63	–	–	30	41
20	*E_coli (ATP)*	–	–	–	–	–	–	33	44	30	43
21	*C_bur (ATP)*	–	–	–	–	–	–	27	41	26	41
22	*A_tha (ATP)*	–	–	–	–	–	–	27	44	32	41
23	*A_suc (ATP)*	–	–	–	–	–	–	22	44	24	39
24	*V_chl (ATP)*	–	–	–	–	–	–	25	46	25	49
25	*Z_may (ATP)*	–	–	–	–	–	–	25	44	29	45
26	*S_one (ATP)*	–	–	–	–	–	–	25	42	25	53
27	*H_inf (ATP)*	–	–	–	–	–	–	29	47	32	44
28	*S_aur (ATP)*	–	–	–	–	–	–	26	43	26	38
29	*W_glo (ATP)*	–	–	–	–	–	–	25	44	36	48
30	*S_cer (ATP)*	–	–	–	–	–	–	27	43	29	44
31	*P_gin (ATP)*	–						25	45	29	43
32	*C_sat (ATP)*	–	–	–	–	–	–	24	40	30	38
33	*L_cas*	–	–	–	–	–	–	25	43	24	40
34	*S_bov*	–	–	–	–	–	–	25	40	24	43

**Table 2.** Alignment of the active site residues including the PCK-specific region, Kinase 1a/P-loop, kinase-2 region and nucleotide binding sites of few selected PCKs. Active site residues are shown in red, mismatches are in blue.

**Species**	**PCK-specific domain**	**Kinase-1a (P-loop)**	**Kinase-2**	**Nucleotide binding region**
*A_suum*	YGGNSLLGKK	SACGKTN	VIGDD	WFRQSA-DHKFLWPGY
*H_con*	YGGNSLLGKK	SACGKTN	CVGDD	WFRRDA-NNKFLWPGY
*N_nor*	YGGNSLLGKK	SAC-ATN	CVGDD	WFRKVK-KGRFIWPGF
*L_van*	YGGNTLLGKK	SACGSTN	CVGDD	WFRKDE-KARFIWPGF
*C_gra*	YGGNSLLGKK	SACRKTN	CVGDD	WFRKNE-KGRFKGPGF
*D_mel*	YGGNSLLGKK	SACGKTN	CVGDD	WFRKSA-EGKFMW*PGY*
*H_sap*	YGGNSLLGKK	SACGKTN	CVGDD	WFRKSA-EGKFMWPGY
*P_tro*	YGGNSLLGKK	SACGKTN	CVGDD	WFRRDE-AGHFLWPGF
*C_dip*	YGGNAILAKK	SACGKTN	VVGDD	WFRRGD-DGRFLWPGF
*C_glu*	YGGNAILAKK	SACGKTN	VVGDD	WFRRGE-DGRFLWPGF
*M_tub*	YGGNALLGKK	SACGKTN	TLGDD	WFRRGD-DGRFLWPGF
*S_ave*	YGGNALLGKK	SACGKTN	TIGDD	WFRKND-EGKFVWPGF
*N_fro*	YGGNALLGKK	SACGKTN	CVGDD	WFRKD–NGRFLWPGY
*T_pal*	YGGNALLGKK	SACGKTN	TVGDD	WFRKDA-EGNFLWPGY
*C_pne*	YGGNALLGKK	SACGKTN	CIGDD	WFRKNN-QGEFLWPGF
*T_aci*	YGGNALLSKK	SASGKTN	LLSDD	WFRRRQ-DGSFIWPGF
*T_vol*	YGGNALLSKK	SASGKTN	LISDD	WFRRRA-DGTFIWPGF
*P_fur*	YGGNVIGLKK	SMCGKTS	IVGDD	YFLRE–NGQWLNEKLD
*T_kod*	YGGNTIGLKK	SMCGKTS	IVGDD	YFLRE–NGVWLNHKLD
*G_int*	YAGNALACKK	SACGKTS	IIGDD	YFLKDPTTGEYLNSKLD
*Z_may*	YAGE—MKK	SGTGKTT	LIGDD	YGVG–KRIRLPYTR
*A_tha*	YAGE—MKK	SGTGKTT	LIGDD	YGTG–SRIKLAYTR
*C_sat*	YAGE—MKK	SGTGKTT	LIGDD	YGSG–NRIKLAYTR
*S_cer*	YAGE—MKK	SGTGKTT	LIGDD	YVSGG-KRCPLKYTR
*B_mar*	YAGE—MKK	SGTGKTT	LIGDD	YGVG–SRFKLKYTR
*P_fal*	YAGE—MKK	SGTGKTT	LIGDD	YGSDNGIRIPLKYTR
*T_cru*	YAGE—MKK	SGTGKTT	LIGDD	RADRGAKRMPLRVTR
*E_coli*	YGGE—MKK	SGTGKTT	LIGDD	WNGTG-KRISIKDTR
*V_chl*	YGGE—MKK	SGTGKTT	LIGDD	WNGSG-KRISIKDTR
*H_inf*	YGGE—MKK	SGTGKTT	LIGDD	WNGTG-KRISIKDTR
*P_gin*	YGGE—MKK	SGTGKTT	LIGDD	WNGTG-KRISIKDTR
*W_glo*	YGGE—IKK	SGTGKTT	LIGDD	WNGKR-ERYSLEYTR
*C_bur*	YAGE—MKK	SGTGKTT	LIGDD	WTGGAEERFSIPTTR
*S_one*	YAGE—MKK	SGTGKTT	LIGDD	WTGGIGKRFDIPTTR
*S_ace*	YAGE—MKK	SGTGKTT	LIGDD	WTGGKYRRISLHYTR
*S_bov*	YFGE—LKK	SGSGKST	VLHDD	YLG—QSIPKEVTL
*L_cas*	YFGE—LKK	SGSGKST	VLHDD	YNG—QNVKPADTL
*C_stu*	YYGE—SKK	SGTGKTT	ILQDD	GKKVTK-RKVKRVEI
*A_per*	YYGE—LKM	SGTGKTT	VMQDD	HAKD—RKIPPELS

**Table 3.** Score values from ClustalW between PCK genes from all domains of life.

**Oraganism**	***D_mel***	***C_gra***	***T_vol***	***A_per***	***C_stu***
*D_mel*	100	0	3	1	3
*C_gra*	0	100	30	1	1
*T_vol*	3	30	100	2	1
*A_per*	1	1	2	100	10
*C_stu*	3	1	1	10	100
*A_suu*	0	46	30	1	1
*H_con*	1	48	30	1	1
*N_fro*	2	37	33	1	2
*H_sap*	1	57	30	2	1
*P_fal*	2	1	3	1	1
*G_inf*	1	3	7	1	1
*P_fur*	1	4	34	4	2
*E_coli*	2	1	1	2	1
*T_kod*	1	30	6	1	1
*C_bur*	1	1	1	3	3
*A_tha*	2	1	0	1	1
*C_glu*	1	44	37	1	1
*A_suc*	2	1	1	1	3
*V_chl*	1	1	0	2	3
*Z_may*	1	1	1	2	2
*S_one*	1	1	1	2	1
*H_inf*	4	1	1	1	3
*S_aur*	5	1	2	2	1
*S_ave*	3	58	35	1	1
*T_aci*	1	32	69	1	2
*W_glo*	1	1	2	1	1
*S_cer*	1	1	0	2	2
*P_tro*	0	51	12	1	1
*B_mar*	0	2	1	1	5
*P_gin*	1	1	2	1	3
*C_sat*	0	1	1	3	4
*L_cas*	1	1	1	1	1
*S_bov*	1	1	1	1	4

**Table 4:**
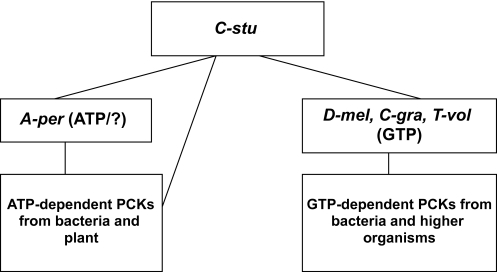
PCK evolution flowchart.
